# Linezolid resistance in patients with drug-resistant TB

**DOI:** 10.5588/ijtld.22.0632

**Published:** 2023-07-01

**Authors:** D. Vengurlekar, C. Walker, R. Mahajan, A. Dalal, V. Chavan, M. A. Galindo, A. Iyer, H. Mansoor, A. Silsarma, P. Isaakidis, H. Spencer

**Affiliations:** 1 Médecins Sans Frontières (MSF) Operational Centre Brussels, Mumbai, India; 2 MSF Operational Centre Brussels, Southern Africa Medical Unit, Cape Town, South Africa; 3 MSF Luxembourg Operational Research Unit (LuxOR), Luxembourg, Luxembourg; 4 MSF Operational Centre Barcelona-Athens, New Delhi, India; 5 Jupiter Hospital, Mumbai, India; 6 Clinical and Molecular Epidemiology Unit, Department of Hygiene and Epidemiology, University of Ioannina School of Medicine, Ioannina, Greece

Dear Editor,

Multidrug-resistant TB (MDR-TB) continues to be a global public health issue. Linezolid (LZD) has been shown to be one of the most effective drugs against MDR-TB.^1^ A meta-analysis of 12,030 patients showed treatment success was positively associated with LZD use (adjusted risk difference 0.15, 95% confidence interval [CI] 0.11–0.18) compared to not using the drug.^2^ New treatment regimens containing bedaquiline (BDQ), pretomanid, LZD with or without moxifloxacin (BPaLM/BPaL) have been recommended by the WHO for MDR-TB programmes.^3^ Unfortunately, global resistance to LZD has been observed, especially in India, which has a high burden of MDR-TB.^4^–^6^ Potential risk factors to acquired LZD resistance are addition of LZD to a failing or inadequate regimen, or interruption of LZD due to adverse events or loss to follow-up.^7^ In a recent meta-analysis, pooled frequency of LZD resistance in clinical isolates of MDR-TB bacteria was reported to be 4.2%.^4^ However, the majority of the studies included in this analysis were from China and Turkey, with only one carried out in India.^4^ Here we report on the clinical/epidemiological profile and treatment outcome of patients with LZD resistance admitted to a Médecins Sans Frontières (MSF) clinic in Mumbai, India.

This was a retrospective, cohort study using routinely collected clinical data from 1^st^ January 2016 to 31^st^ December 2020. Mumbai is one of India’s most populous cities, and has a high prevalence of drug-resistant TB (DR-TB).^5^,^8^ A cohort of patients who had failed DR-TB treatment regimens were retreated in the MSF Clinic in Mumbai’s M East Ward. Before starting treatment, patient files were reviewed by a DR-TB Technical Expert Committee. Drug susceptibility testing (DST) and prior exposure to anti-TB medications were used to customise treatment plans. Previously treated patients on failing MDR-TB regimens were provided treatment at the Clinic. Culture-based phenotypic DST using Mycobacteria Growth Indicator Tube (BD, Franklin Lakes, NJ, USA) in modified Middlebrook 7H9 broth medium was performed for second-line injectable agents (kanamycin, amikacin, capreomycin) ethionamide, para-aminosalicylic acid, clofazimine, LZD, BDQ and the fluoroquinolones (ofloxacin, levofloxacin and moxifloxacin). Prior to enrolment at the clinic, patients’ laboratory investigations and follow-up included GeneXpert testing (Cepheid, Sunnyvale, CA, USA), first-line and second-line line-probe assays, culture-based DST, chest radiographs (CXRs) and other relevant radiological examinations. Treatment lasted 20–22 months. A multidisciplinary team provided clinical and psychosocial support. Patients were followed up every month after enrolment and monthly sputum culture was done once treatment began. Treatment outcomes were defined according to national guidelines (cured, completed, failed, death, lost to follow-up).^9^ Unfavourable outcomes were defined as treatment failure or died. Risk factors for unfavourable treatment outcome were tested using multivariable logistic regression; risk factors with *P* < 0.2. in univariate analysis were included in the model. Cumulative incidence of the unfavourable treatment outcome was estimated using the Kaplan–Meier method.

Between 2016 and 2020, 365 DR-TB patients were registered and LZD resistance was found in 19.7% (72/365). The median age of patients with LZD resistance was 28 years (interquartile range [IQR] 22–35); 53% (38/72) were male; 39% (28/72) were severely underweight (BMI-for-age *Z*-score of −3 for adolescents aged 11–17 years and a BMI of 16.5 kg/m^2^ in adults), and 7% (5/72) had extrapulmonary TB. At the time of enrolment, respectively 8% (6/72), 7% (5/72), 1.4% (1/72) and 1.4% (1/72) of patients had peripheral neuropathy, hepatitis B, HIV and diabetes mellitus. Most (85%, 61/72) had received DR-TB treatment in the past. Three quarters of patients (78%, 56/72) had previously been treated with LZD for a median duration of 18 months (IQR 8–23). Exposure history to LZD was unknown for the 16 remaining cases. Patients were treated with a combination of BDQ, delamanid and imipenem (*n* = 53), BDQ and delamanid (*n* = 11) and other regimens (*n* = 7), with a favourable outcome percentage of respectively 64% (34/53), 91% (10/11) and 14% (1/7). One patient died before initiating treatment. The median treatment length for BDQ and delamanid was 18 months (IQR 8–19), and 7 months (IQR 5–9) for imipenem. In the multivariable model, only *Mycobacterium tuberculosis* in culture and diabetes mellitus at baseline were significantly associated with unfavourable treatment outcomes. Although male sex (adjusted odds ratio [aOR] 3.5, 95% CI 1–16); *P* = 0.053) and severely underweight status (aOR 3.9, 95% CI 0.5–24; *P* = 0.2) at baseline showed a strong association with unfavourable outcomes, they were not statistically significant.

From the patient cohort, 46% (33/72) were cured, 17% (12/72) completed treatment, 17% (12/72) had treatment failure, 18% (13/72) died and 1.4% (1/72) lost to follow-up. One patient was continuing treatment at time of analysis. The median time to death and treatment failure was respectively 3 (IQR 2–4) and 14 (IQR 11–19) months. Kaplan–Meier estimates showed that the cumulative probability of mortality at 3, 6, 12 and 24 months following treatment initiation was respectively 7%, 16.8%, 18.2% and 18.2%. Cumulative probability of treatment failure at 6, 12 and 24 months was respectively 0%, 7% and 42% (Figure). Of the total 72 patients, sputum samples of six patients were subjected to BDQ DST and were found to be susceptible.

**Figure:**
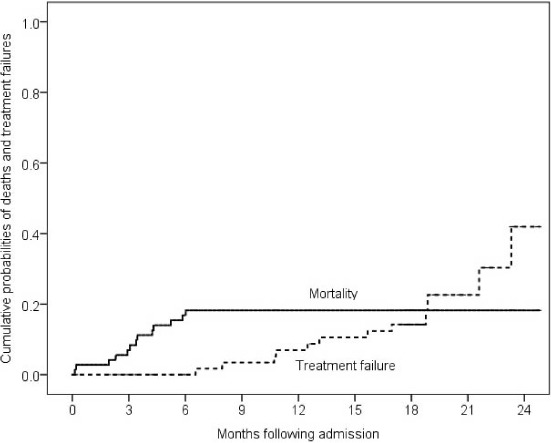
Kaplan–Meier estimates of treatment failure.

In this study, we describe one of the largest cohorts of LZD-resistant patients from India. The extensive resistance profiles in this cohort leave physicians with limited options for effective treatment regimens.^10^,^11^ Over a third (36%) of patients had an unfavourable outcome, compared to 13% across India and 19% in Maharashtra.^12^ This decline in treatment success is likely due to the failure of the previous MDR-TB regimens and the advanced resistance patterns of patients in our cohort.

It is important to thoroughly assess previous LZD exposure and proactively manage adverse events to avoid treatment interruptions, which can contribute to resistance in patients with MDR-TB.^13^ LZD resistance was found in 22% (16/72) of the study population who did not have documented exposure to LZD. This suggests that the patients acquired LZD resistance from strains that develop in the community as a consequence of crowded living conditions, high population density and high MDR-TB prevalence.^14^ Sequencing of resistant strains is needed to better understand this. The emergence of LZD resistance highlights the limited options for treating extensively drug-resistant TB. Multiple retreatment episodes, coupled with an exposure history to LZD, pose significant challenges for MDR-TB treatment. Individualised regimens with newer medications, adequate management of related adverse effects, early detection of LZD resistance using DST and sequencing, and stronger indicators of treatment failure would contribute to better outcomes for TB patients with LZD resistance.
